# Absence of instabilities and intra-prosthetic dislocations at 7 to 11 years following THA using a fourth-generation cementless dual mobility acetabular cup

**DOI:** 10.1186/s40634-020-00265-3

**Published:** 2020-07-13

**Authors:** Julien Chouteau, Jean-Charles Rollier, Michel P. Bonnin, Mo Saffarini, Luca Nover, Jean-Christophe Chatelet, Laurent Jacquot

**Affiliations:** 1 Artro Group Institute, Lyon, France; 2 Clinique d’Argonay, Ramsay Santé, Annecy, France; 3 Centre Orthopédique Santy, Hôpital Privé Jean Mermoz, Ramsay Santé, Lyon, France; 4 ReSurg SA, Nyon, Switzerland; 5 Centre de Chirurgie Orthopédique du Beaujolais, Ramsay Santé, Arnas, France

**Keywords:** Clinical and radiographic outcomes, Dual-mobility acetabular cup, Cementless THA, Dislocations, Survival, Mid-term

## Abstract

**Purpose:**

Dual-mobility (DM) cups are increasingly used in total hip arthroplasty (THA) but there lacks literature on their long-term results. We aimed to investigate outcomes of a fourth-generation cementless DM acetabular cup at 7–11 years.

**Methods:**

We retrospectively evaluated 240 consecutive hips that received cementless THA using the same dual mobility cup (Novae Sunfit TH) and femoral stem (Corail). Patients were recalled at ≥7 years to collect Oxford hip scores (OHS), Harris hip scores (HHS), and inspect for radiolucent lines and granulomas. Multi-variable analyses were performed to determine whether HHS or OHS were associated with pre- or intra-operative variables.

**Results:**

At 8.4 ± 0.8 years (range, 7–11), 6 hips were revised (2.5%), 54 deceased (22.5%), and 14 could not be reached (5.8%). Four revisions (2 cup+stem, 2 liners only) were due to sepsis (1.7%), one (cup and stem) for trauma (0.4%), and one (stem) due to aseptic loosening (0.4%). For the remaining 166 hips, HHS was 83.6 ± 13.2 and OHS was 20.3 ± 6.7. Multi-variable analysis confirmed that HHS (β = − 0.38; *p* = 0.039) and OHS (β = 0.36; *p* < 0.001) worsened with age, and that OHS was worse for Charnley C patients (β = 3.17; *p* = 0.009). Neither granulomas nor radiolucenies were observed around any cups, but radiolucenies were seen around 25 stems (20.3%).

**Conclusions:**

This fourth-generation DM cup demonstrated satisfactory outcomes at 7–11 years, with no instabilities or cup revisions due to aseptic loosening. Better OHS was observed for younger patients and those presenting higher Charnley grade.

**Level of evidence:**

Level IV, retrospective case study.

## Background

Dislocation after total hip arthroplasty (THA) is a burdensome complication, observed in up to 10% of cases [2, 23, 36, 38], though one must consider heterogeneity among studied population, follow-up, and other confounding factors. Dual mobility (DM) cups became increasingly popular in recent years, as they proved effective at preventing articular instability, by virtue of increased ‘jump distance’ and ratio of head-to-neck diameter [25, 47]. Though the initial ‘Bousquet’ cup was prone to intra-prosthetic dislocations (IPD) and aseptic loosening [37, 40, 42, 46], design improvements over the past decades resolved many shortcomings of DM cups, thanks to enhanced press-fit and bioactive coatings [10, 31], as well as more durable liners made of ultra-high molecular weight polyethylene (UHMWPE) [37, 45].

Fourth-generation DM cups have proved effective at preventing IPD [11, 34, 46] and demonstrated promising complication and survival rates [18, 38, 39]. While originally intended for patients at risk of subluxation and dislocation, notably geriatric patients [1, 3] and those with femoral neck fractures [3, 24, 27, 28, 33, 41, 46] or neuromuscular deficit [9, 49], DM cups are increasingly used in younger and more active cohorts [31, 40, 49]. Their mid- to long-term outcomes yet are scarcely documented and could reassure clinicians worldwide of their benefits and suitability for a wider range of indications [2, 25, 33].

The primary goal of this study was to report revision rates, clinical scores and radiologic findings of a fourth-generation DM acetabular cup, in a sizeable multi-centre series with up to ten years of follow-up. The secondary goal was to identify demographic and operative factors that could compromise clinical scores and hence optimise future patient selection and surgical choices.

## Methods

**Fig. 1:**
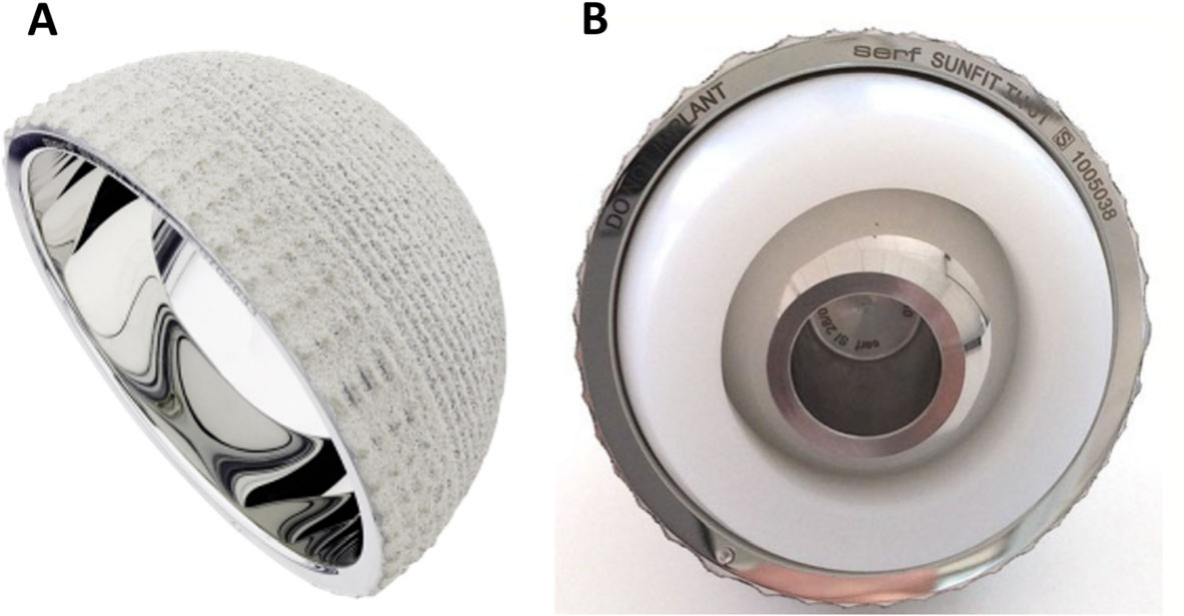
The Novae Sunfit TH cementless dual mobility cup: (**a**) metallic shell with mirror-polished articular surface and rough porous outer surface coated with titanium and HA; (**b**) femoral head assembled with the retentive PE mobile insert that articulates within the metallic shaft

This study was prospectively designed prior to collecting data on retrospectively operated patients. The authors evaluated a consecutive series of 240 THAs (225 patients) performed over three consecutive years (June 2007 to June 10) using the same cementless dual mobility cup (Novae Sunfit TH, Serf, Décines, France) (Fig. 1) withthe same femoral stem (Corail, Depuy, Leeds, UK) by 3 surgeons (LJ, JCR, JCC). The femoral heads used were made of ceramic (*n* = 164) or metal (*n* = 76), and were of diameter 28 mm (*n* = 238) or 22 mm (n = 2). All implants had been approved and in routine clinical use before the inclusion period. The cohort comprised 81 men (93 hips) and 129 women (147 hips), aged 77.4 ± 5.6 years (range, 54–94), with body mass index (BMI) of 26.6 ± 4.6 (range, 17.9–40.6), ASA score of 2 ± 1 (range, 1–3) (Table 1). Preoperative walking ability was assessed using the Charnley classification [43]. The etiology was primary osteoarthritis for 207 hips (87%), avascular necrosis for 18 hips (8%), and secondary osteoarthritis for 15 hips (6%). The procedures were performed through a posterior approach for 169 hips (70%) and anterolateral approach in 71 hips (30%). The mean cup size (diameter) used was 52.4 ± 3.0 (range, 47–63).

**Table 1:** Preoperative demographics, and morphological data

	Original Cohort			
	(*n* = 240 hips)			
	Mean	±SD	Range	
Age	77.4	± 5.6	(54.0	- 94.0)
BMI	26.6	± 4.6	(17.9	- 40.6)
ASA score	2	± 1	(1	- 3)
Stem Size	12	± 2	(8	- 16)
Cup Size	52	± 3	(47	- 63)
Male gender	93	(39%)		
Bilateral cases	15	(6%)		
Charnley grade				
A	147	(61%)		
B	26	(11%)		
C	66	(28%)		
Missing	1	(0%)		
Etiology				
Primary OA	207	(86%)		
Secondary OA	15	(6%)		
Avascular necrosis	18	(8%)		
Surgical Approach				
Posterior	169	(70%)		
Anterolateral	71	(30%)		
Stem type				
KA - Standard	133	(55%)		
KHO - High offset	17	(7%)		
KLA - Lateralized	90	(38%)		

All patients were recalled for clinical and radiographic evaluation, and their case notes were used to document implant materials, models and diameters. From the initial cohort of 240 THAs, 3 had stem and cup revisions, 2 had liner and/or head replacement, and 1 had an isolated stem revision. In addition, 50 patients (54 hips) deceased, none of which had revision surgery, and 14 patients (14 hips) could not be contacted. The remaining 166 hips were assessed clinically at 8.4 ± 0.8 years (range, 7–11), of which 123 hips were also assessed radiographically. The clinical scores collected included the Oxford hip score (OHS, best = 12; worst = 60) [13], Harris hip score (HHS, best = 100; worst = 0) (best) [5], pain on visual analogic scale (pVAS, best = 0; worst = 10). The radiographic assessment included frontal weight-bearing pelvic radiographs that were inspected for radiolucent lines (> 2 mm wide) and granulomas in the 7 femoral Gruen zones [19] and 3 acetabular DeLee–Charnley zones [6, 14], and for Brooker heterotopic ossifications [4]. All patients provided written informed consent for their participation in the study.

### Statistical analysis

Normality of distributions was verified using the Shapiro–Wilk test. In case of non-parametric quantitative data, significance of differences between groups was assessed by the Mann–Whitney U test (Wilcoxon rank-sum test). Uni- and multi-variable linear regression analyses were performed after identification of relevant variables (age at surgery, gender, BMI, indications, Charnley grade, surgical approach, stem size and type and cup sizes), by backward selection using the threshold *p* = 0.15, to determine their associations with 2 main outcomes (HHS and OHS). Statistical analyses were performed using R version 3.5.2 (R Foundation for statistical computing, Vienna, Austria). *P* values < 0.05 were deemed statistically significant

## Results

**Fig. 2:**
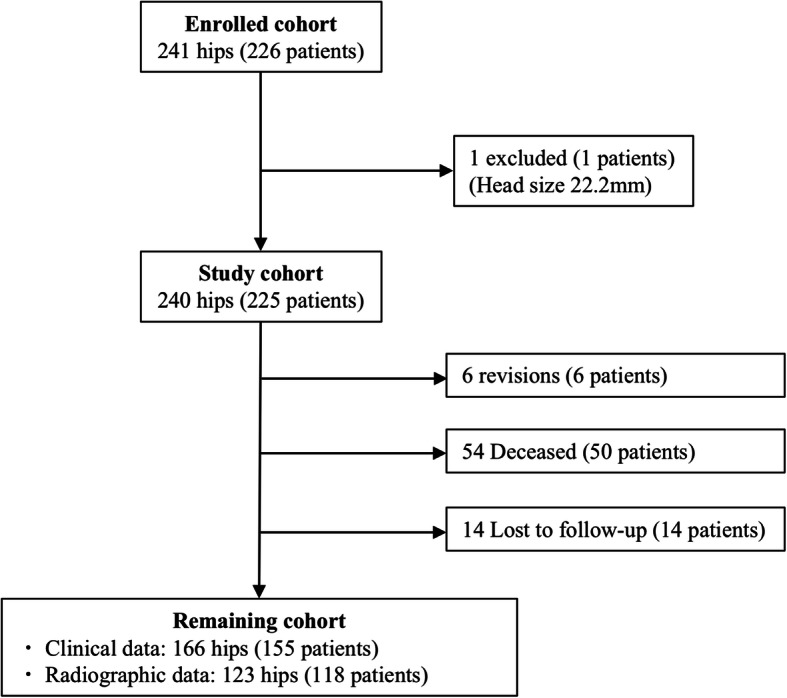
Flowchart indicating numbers of hips (patients) in the enrolled cohort, exclusions, revisions and losses

**Fig. 3:**
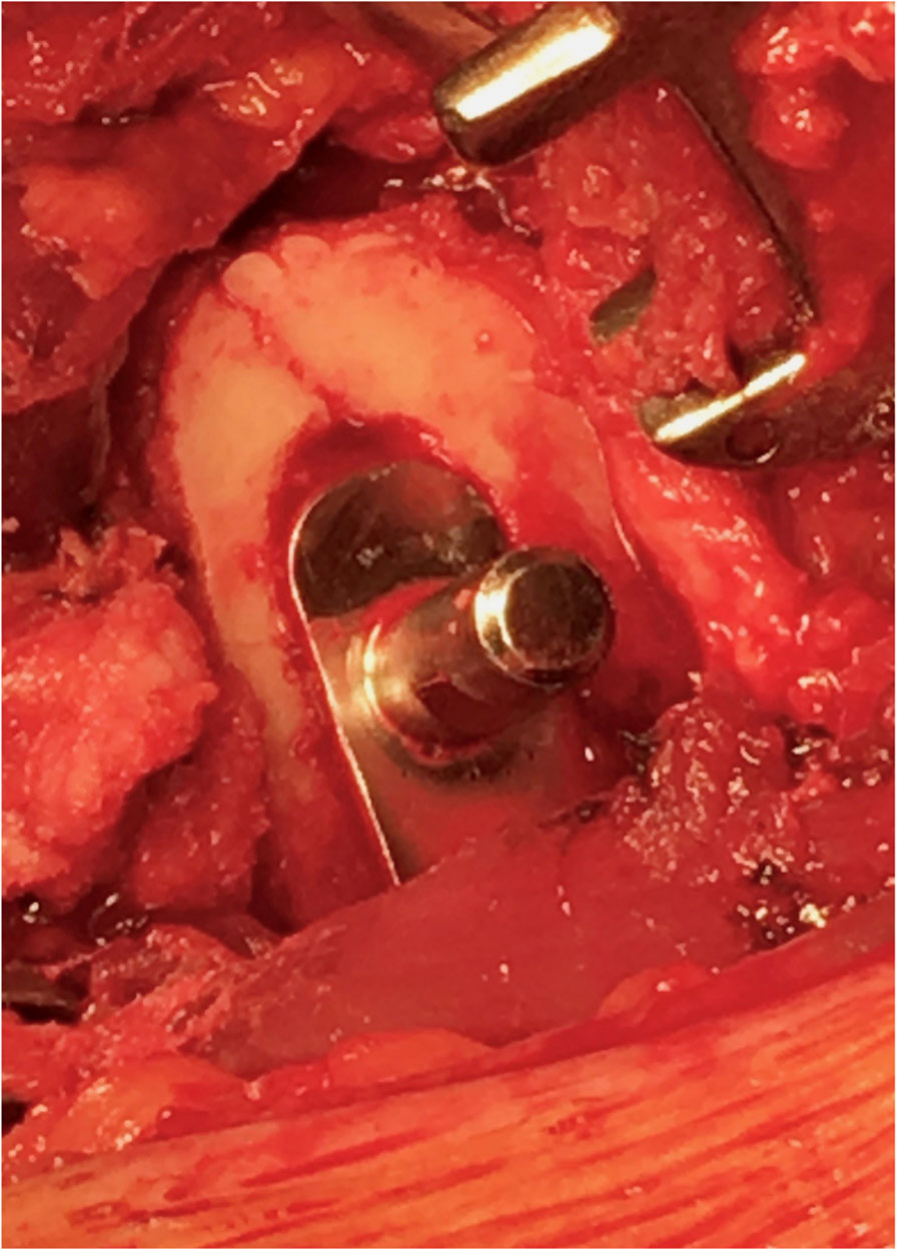
Intraoperative femoral crack observed during broaching which was fixed using a cerclage wire

From the cohort of 225 patients (240 hips), 6 patients (6 hips, 2.5%) were revised, 50 patients (54 hips, 22.5%) died, and 14 patients (14 hips, 5.8%) were lost to follow-up (Fig. 2). Four of the revisions were due to deep infection (1.7%), 2 of which required cup and stem exchange (0.8%) while 2 required only PE liner exchange (0.8%). One of the revisions was due to periprosthetic femoral fracture secondary to trauma which required cup and stem exchange (0.4%), and only one revision was due to aseptic loosening and required isolated stem revision (0.4%). There were no dislocations recorded. Furthermore, there were 21 complications that did not require revision (8.8%), including 2 deep infections treated by lavage (0.8%), as well as 12 intraoperative femoral cracks (5%), all observed during broaching (Fig. 3) and resolved using cerclage wires, 3 of which later developed iliopsoas impingements (1.3%).

### Clinical outcomes

For the final cohort of 155 patients (166 hips) who still have their original implants in place the HHS improved from 41.7 ± 13.1 (range, 10–74) preoperatively, to 83.6 ± 13.2 (range, 8–99) postoperatively (Table 2). Their OHS was 20.3 ± 6.7 (range, 12–42) and pVAS was 0.6 ± 1.3 (range, 0–7). Uni-variable analysis revealed that HHS worsened with patient age (β = − 0.40*p* = 0.030) (Table 3) and that OHS worsened with patient age (β = 0.34; *p* < 0.001) and was worse for patients with Charnley C walking ability (β = 3.28; *p* = 0.009) (Table 4). Multi-variable analysis confirmed that HHS and OHS worsened with age (respectively, β = − 0.38; *p* = 0.039 and β = 0.36; *p* < 0.001) and that OHS was worse for patients with Charnley C walking ability (β = 2.87; *p* = 0.017).

**Table 2:** Clinical data of the final cohort

	Final cohort (***n*** = 166 hips)			
	Mean	±SD	Median	Range
**Follow-up (yrs)**	**8.4**	**± 0.8**	**8**	**(7 to 11)**
**Pre-op HHS Total**	**41.7**	**± 13.1**	**42**	**(10 to 74)**
Pain	10.3	± 7.7	10	(0 to 30)
Function	25.1	± 7.6	26	(0 to 42)
Mobility	2.0	± 0.3	2	(1 to 3)
Attitude	3.9	± 0.3	4	(2 to 4)
**Post-op HHS Total**	**83.6**	**± 13.2**	**86**	**(8 to 99)**
Pain	41.0	± 6.7	44	(2 to 44)
Function	35.2	± 9.9	37	(0 to 47)
Mobility	3.2	± 0.6	3	(0 to 5)
Attitude	4.0	± 0.2	4	(3 to 4)
**HHS Total Improvement**	**41.5**	**± 17.7**	**42**	**(−35 to 76)**
Pain	30.9	± 10.5	34	(−10 to 44)
Function	10.9	± 11.6	11	(−31 to 42)
Mobility	1.1	± 0.7	1	(−2 to 3)
Attitude	0.0	± 0.3	0	(−1 to 2)
**Post-op OHS**	**20.3**	**± 6.7**	**19**	**(12 to 42)**
**Post-op Pain on VAS**	**0.6**	**± 1.3**	**0**	**(0 to 7)**
**Devane activity grade**				
1	20	(12%)		
2	56	(34%)		
3	61	(37%)		
4	21	(13%)		
5	4	(2%)		
na	4	(2%)

**Table 3:** Uni- and multi-variable regression analysis of Harris Hip Score

Variable	Univariable			Multivariable (***n*** = 157)*		
	β	95% C.I.	***p***-value	β	95% C.I.	***p***-value
**Preoperative data**						
Age at index operation (yrs)	−0.40	(− 0.76 – –0.04)	**0.030**	−0.38	(− 0.74 – –0.02)	**0.039**
Body Mass Index (BMI)	− 0.12	(− 0.59 – 0.35)	0.603			
Male sex	1.41	(−2.88 – 5.71)	0.517			
Etiology						
*Primary arthrosis*	REF					
*Secondary arthrosis*	0.93	(−6.95 – 8.81)	0.816			
*Avascular necorsis*	3.07	(−4.53 – 10.66)	0.427			
Charnley grade						
*A*	REF			REF		
*B*	1.88	(−5.49 – 9.24)	0.615	1.25	(−6.03 – 8.53)	0.735
*C*	−4.89	(9.94 – 0.15)	0.057	−4.76	(−9.73 – 0.21)	0.061
**Intraoperative data**						
Stem size	−0.47	(−1.81 – 0.87)	0.491			
Cup size	− 0.01	(− 0.75 – 0.73)	0.976			
Stem type						
*Standard - KA*	REF					
*High offset - KHO*	4.10	(−3.72 – 11.91)	0.302			
*Lateralized - KLA*	1.84	(−2.52 – 6.20)	0.406			
REF						
−2.25		(−6.88 – 2.37)	0.338			

*Backward selection (*p* = 0.15) was used to identify variables to include in the multivariabla analysis

**Table 4:** Uni- and multi-variable regression analysis of Oxford Hip Score

Variable	Univariable			Multivariable (***n*** = 163)*		
	β	95% C.I.	***p***-value	β	95% C.I.	***p***-value
**Preoperative data**						
Age at index operation (yrs)	0.34	(0.16 – 0.51)	**< 0.001**	0.36	(0.18 – 0.53)	**< 0.001**
Body Mass Index (BMI)	−0.12	(−0.35 – 0.12)	0.325			
Male sex	−0.49	(−2.64 – 1.66)	0.653			
Etiology						
*Primary arthrosis*	REF					
*Secondary arthrosis*	0.47	(−3.37 – 4.32)	0.808			
*Avascular necorsis*	−2.37	(−6.22 – 1.47)	0.225			
Charnley grade						
*A*	REF			REF		
*B*	2.56	(−0.93 – 6.05)	0.150	2.95	(− 0.39 – 6.30)	0.083
*C*	3.28	(0.82 – 5.74)	**0.009**	3.17	(0.81 – 5.52)	**0.009**
**Intraoperative data**						
Stem size	0.25	(−0.41 – 0.91)	0.458			
Cup size	0.02	(−0.35 – 0.38)	0.922			
Stem type						
*Standard - KA*	REF					
*High offset - KHO*	−0.46	(−4.44 – 3.52)	0.821			
*Lateralized - KLA*	0.25	(−1.93 – 2.43)	0.821			
Surgical Approach						
*Posterior*	REF			REF		
*Watson-Jones*	−0.42	(−2.71 – 1.88)	0.721	−1.38	(−3.59 – 0.82)	0.216

*Backward selection (*p* = 0.15) was used to identify variables to include in the multivariabla analysis

### Radiographic outcomes

Radiographic assessment was performed for 118 patients (123 hips) for which x-rays were available at final follow-up. We observed heterotopic ossification of grade I in 18 hips (14.6%), grade II in 2 hips (1.6%), and grade III in 1 hip (0.8%). Neither granulomas nor radiolucent lines were observed around any cups, but there were radiolucenies around 25 femoral stems (20.3%): 24 in Gruen zone 1 (19.5%) and 1 in Gruen zone 7 (0.8%).

## Discussion

This study demonstrated satisfactory clinical and radiographic outcomes of cementless THA using a fourth-generation DM acetabular cup, with a cumulative revision rate of 2.5% at a mean follow-up of 8.4 years. It is important to note, however, that only one revision (0.4%) was due to aseptic loosening, and required femoral component exchange, but that there were no cup revisions, due to either instability or aseptic loosening. Deep infection remained the principal cause of revision (1.7%) and only one hip was revised for periprosthetic fracture (0.4%) secondary to trauma. Our cumulative revision rate is within the range reported for fourth-generation DM acetabular cups [7, 8, 16–18, 21, 22, 31, 32, 51, 52]. While numerous smaller series (40–104 hips) [31, 32, 51, 52] had no revisions of any kind at 5 to 10 years of follow-up, larger cohorts (167–3474 hips) [7, 8, 16–18, 21, 22] had overall revision rates between 0.5% and 3.6%, at 5 to 13 years of follow-up.

Dual mobility acetabular cups proved increasingly popular in recent years as they allow improved range of motion and prevent instabilities [25, 47]. Fourth-generation DM acetabular cups, with optimized bearing surfaces, liner materials and coatings, have reduced the risks of intra-prosthetic dislocations and the need for subsequent revisions [37, 40, 42, 46]. However, there still remains a lack of published studies concerning their mid- to long-term outcomes.

Recent studies indicated that deep infection is the most common cause for revision of DM acetabular cups [29], which may be explained by the infirmity and comorbidities of the older population in which they are implanted [3]. In this series, the cumulative rate of revision for infection was 1.7%, at 8.4 years which is slightly higher than the rate of 1.0% at 5 years, reported for all hip arthroplasty infections in the Danish hip registery [20].

This study revealed no intra-prosthetic instabilities at either the liner-cup junction or at the liner-head junction, which proves that 28 mm heads are compatible with this stem and cup combination [11]. According to the current literature, it is clear that DM is the best option to prevent instabilities after THA, particularly in women, elderly and obese patients, as well as those with elevated ASA scores or neuromuscular deficits [3, 35, 47]. Moreover, it is still debateable whether larger femoral head sizes should be used, as they are associated with lower risks of dislocations but increased PE wear [26, 30, 48]. Our study revealed no dislocations using 28 mm heads. Using larger femoral head sizes could exacerbate PE wear, debris and osteolysis [15], whereas using 22 mm heads, would increase the risk of intra-prosthetic instabilities by reducing the neck to head ratio, which causes earlier impingement between the stem neck and the retentive cup rim [12, 44]. Psoas impingement was found in 3 hips (1.3%), all of which had intraoperative femoral cracks fixed using cerclage wires, which likely exacerbated tendon contact against implanted components. In a landmark anatomic study, Vandenbussche et al. [50] described the acetabular zone of psoas impingement, and warned that prosthetic overhang is more frequent with DM acetabular cups, because they are designed with a more protrusive rim.

For the present series, the median HHS and OHS at 7 to 11 years were 86 and 19 points respectively, and patient-repoted pVAS was 0. These outcomes compare favourably to scores repoted in recent studies on fourth-generation DM acetabular cups [7, 8, 18, 21, 32, 51, 52]. Our multi-variable analysis revealed significant influence of preoperative Charnley disability index and age on OHS, both of which are not surprising. Postoperative radiographic analysis revealed absence of radiolucent lines around the acetabular cup, suggesting adequate osteointegration for all cases. Regarding the femur, radiolucent lines could be observed around 25 stems (20%) mostly in Gruen zone 1. No granulomas were noted around the stems or the cups.

The main limitations of the present study are its retrospective design, and hence considerable proportion of patients lost to follow-up (8%) or missing radiographic images (26%). The advanced age of many of the patients may have contributed to the high numbers that were lost to follow-up, but they shared the same standard demographics and surgical parameters as the rest of the series. It is noteworthy that the clinical follow-up was longer than the radiographic follow-up. Despite the size of the initial cohort and follow-up at 7 to 11 years, the present data may be insufficient to confirm elimination of rare complications such as instabilities and intra-prosthetic dislocations, which require larger cohorts with prospective follow-up. National registries provide larger datasets for more robust conclusions on complications and survival but the heterogeneity of implant models and surgical techniques, as well as the paucity of preoperative and surgical data do not enable identification of risk factors. Furthermore, this study is not comparative and cannot therefore decide on the relative functional or cost benefits as compared with unipolar cups. The principal strength of the study is the sizeable cohort, which includes patients susceptible to instabilities, and relatively extended follow-up for a fourth-generation generation of DM acetabular cups. Although two stem head materials were used and two different surgical approaches applied, the same DM acetabular cup design was used throughout the study which allows the authors to draw clear conclusions.

## Conclusion

This study presented satisfactory radiographic and clinical mid-term outcomes of cementless THA using a fourth-generation DM acetabular cup, with no instabilities or revisions due to aseptic loosening. Better HHS and OHS were observed for younger patients and those with preoperative Charnley grade A. Further studies should consider tribologic aspects of DM acetabular cups to confirm the best bearing couples that would minimize wear and metal ion release in the long-term.

## Data Availability

Not applicable.

## Abbreviations

THA: Total Hip Arthroplasty
DM: Dual Mobility
IPD: Intra-Prosthetic Dislocations
UHMWPE: Ultra-High Molecular Weight Polyethylene
BMI: Body Mass Index
OHS: Oxford Hip Score
HHS: Harris Hip Score
pVAS: Pain on Visual Analogic Scale
PE: Polyethylene
